# Supplementary material for: A Spectroscopic and Molecular Dynamics Study on the Aggregation Properties of a Lipopeptide Analogue of Liraglutide, a Therapeutic Peptide against Diabetes Type 2
Source: Molecules. 2023 Nov 11;28(22):7536. doi: 10.3390/molecules28227536 (PMC10674484; doi:10.3390/molecules28227536)
Supplement: Supplementary file 1 [file molecules-28-07536-s001.zip › molecules-2700569-supplementary.pdf]

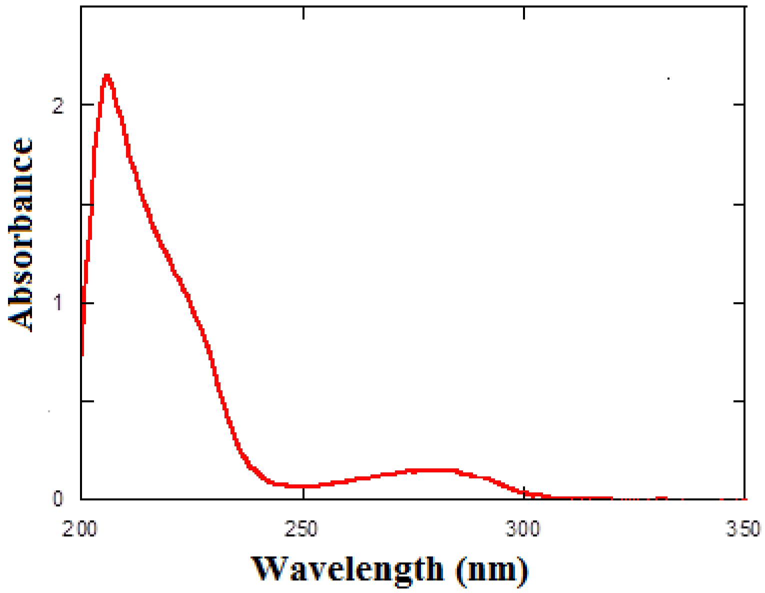

Figure S1. UV absorption spectrum of LG18 in PBS (pH=8.1).

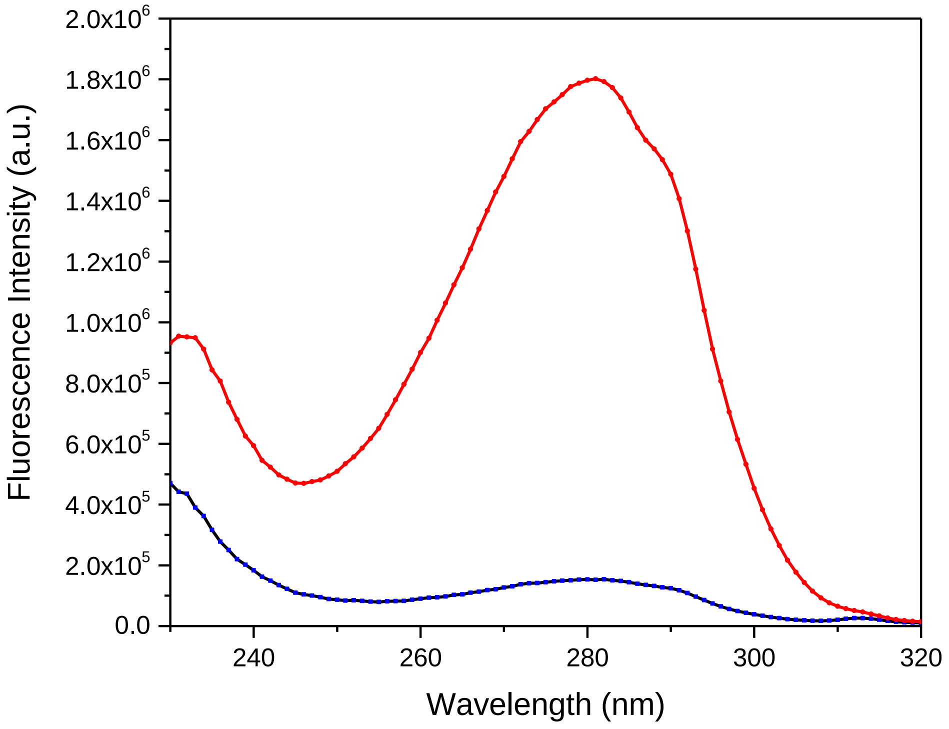

Figure S2. Excitation spectra (λ_{em}=350 nm) of LG18 (21 μM, pH=8). Red: freshly prepared; blue: after two weeks.

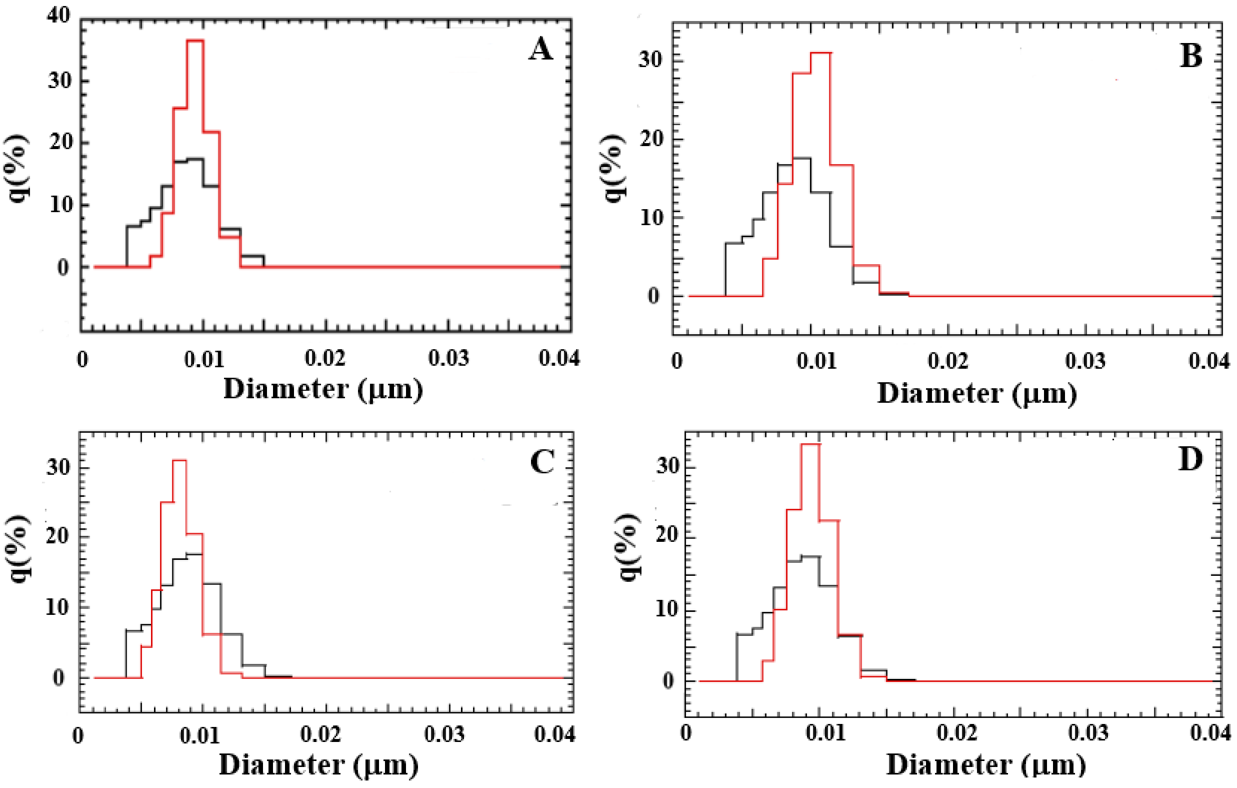

Figure S3. Diffuse light scattering intensity (percent) as a function of the nanoparticle diameter for LG18 solutions. A) red: 0.47 μM; black: pure buffer solution. B) red: 0.94 μM; black: pure buffer solution. C) red: 1.40 μM, black: pure buffer solution; D) red: 2.80 μM, black: pure buffer solution.

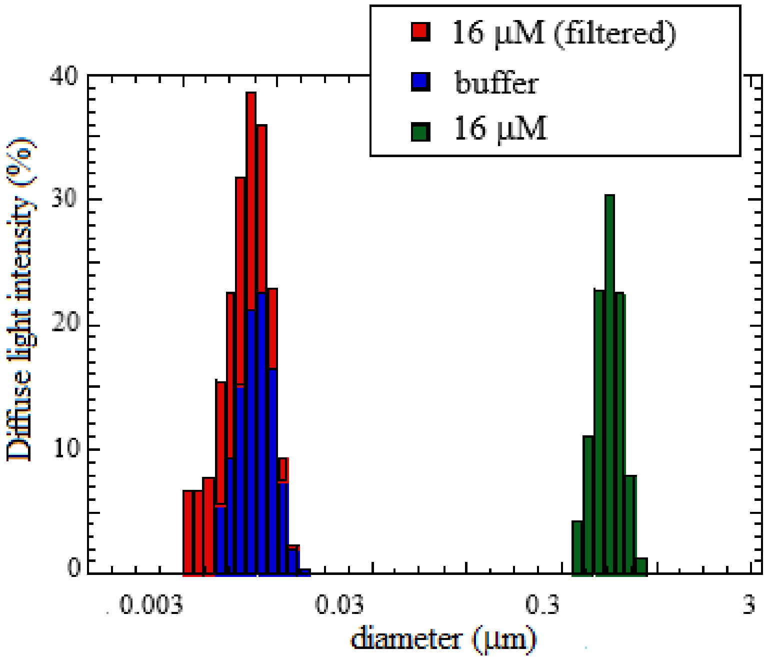

Figure S4. Diffuse light intensity (percent) of 16 µM one-month aged solutions of LG18 (green) as a function of the diameter of the aggregate (nm, log scale). Red: filtered LG18 solution (filter 0.2 µm); blue: phosphate buffer solution.

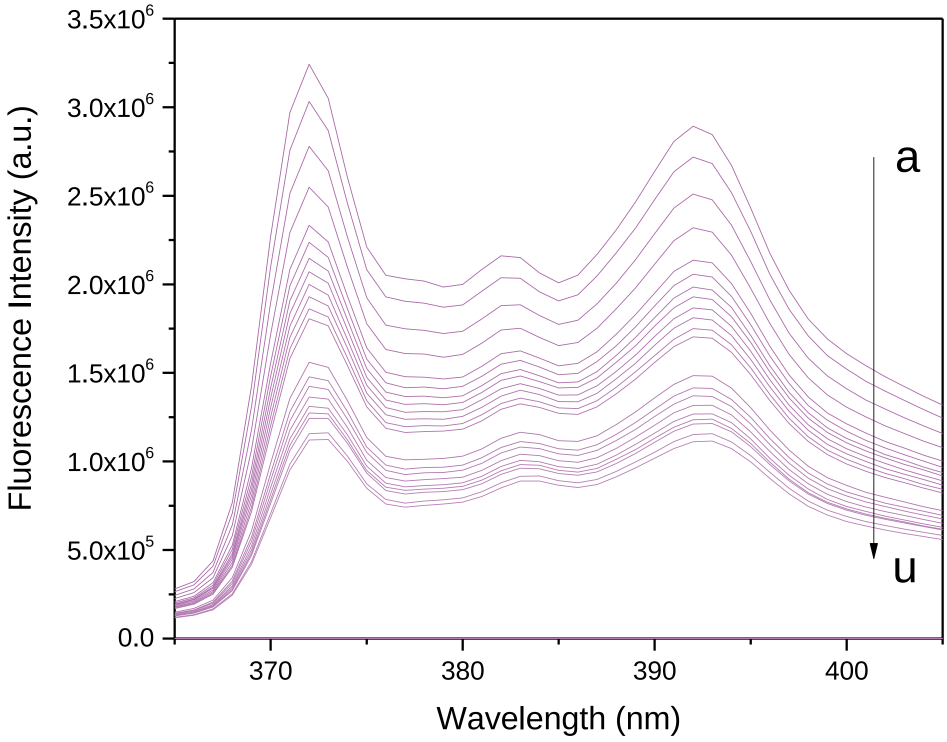

Figure S5. Pyrene (1 µM) emission spectrum (λ_{ex}=319 nm) for different LG18 concentrations: a) 0; b) 0.24 µM; c) 0.47 µM; d) 2.25 µM; e) 4.19 µM; f) 5.88 µM; g) 7.36 µM; h) 8.04 µM; i) 8.67 µM; j) 9.28 µM; k) 10.1 µM; l) 10.7 µM; m) 11.2 µM; n) 11.7 µM; o) 12.9 µM; p) 16.7 µM; q) 17.6 µM; r) 18.3 µM; s) 19.0 µM; t) 20.4 µM; u) 21.0 µM.

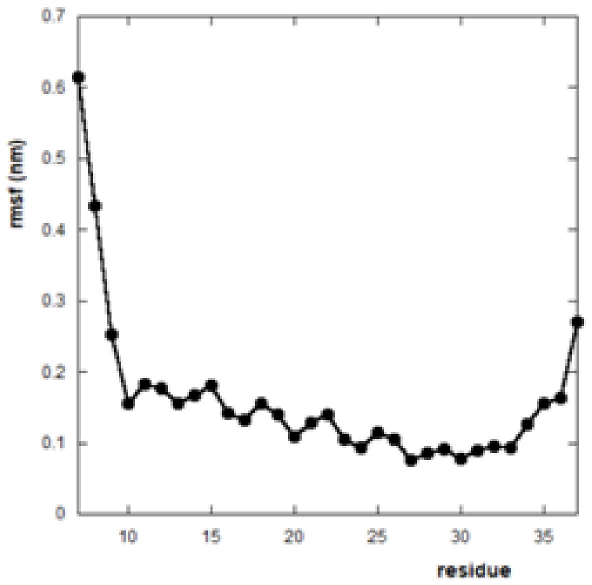

Figure S6. Root means square fluctuations of the C^{α} carbons of the LG18 monomer.

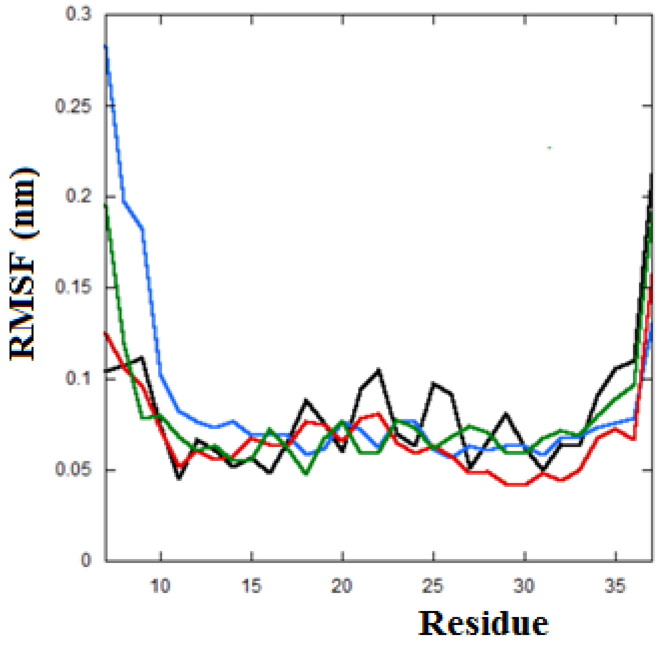

Figure S7. Root means square fluctuations of the C^{α} carbons of the four LG18 chains involved in the formation of an LG18 tetramer.

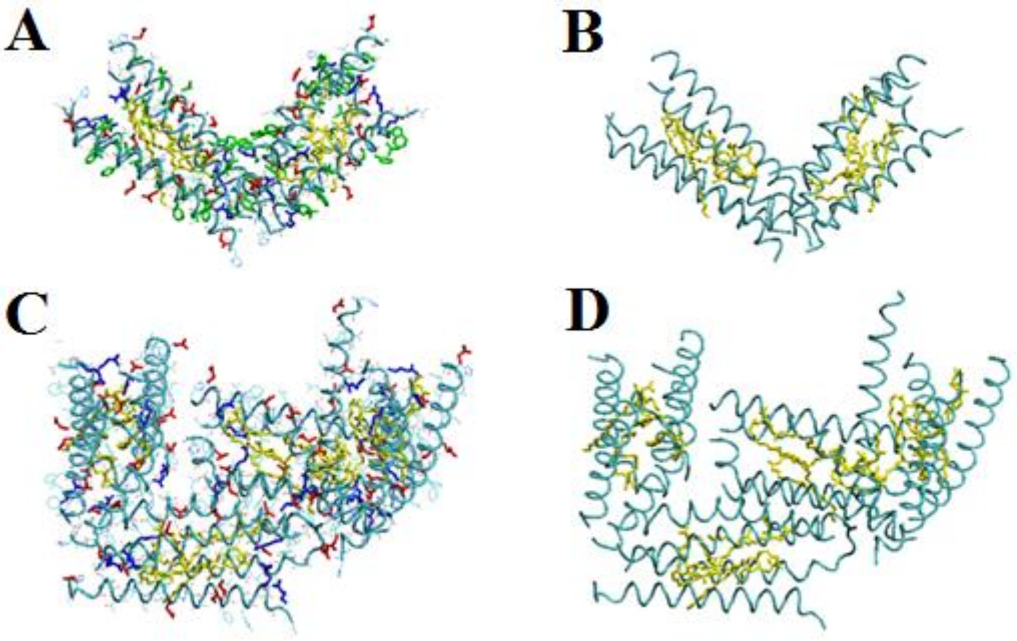

Figure S8. Side view of the aggregates of 8 (A,B) and 16 (C,D) LG18 units after a 100 ns MD simulation. For the sake of clarity, on B and D only the backbones and the K26m side chains are shown. Green: apolar residues; Red: negatively charged residues; Blue: positively charged residues: Yellow: K26m side chains.

Table S1. Time-resolved fluorescence experiments on micromolar LG18 solutions (λ_{ex}=295 nm, λ_{em}=350 nm).

Concentration (μM)	α_1	τ_1 (ns)	α_2	τ_2 (ns)	α_3	τ_3 (ns)	$<\tau>$ (ns)	χ^2
3	0.48	1.07	0.40	3.50	0.12	7.38	2.80	1.25
21	0.45	1.22	0.41	3.66	0.14	7.40	3.00	1.27
36	0.40	1.03	0.43	3.28	0.17	7.11	3.01	1.24

Table S2. Fluorescence intensity ratio of the vibronic components of the pyrene emission at λ_{em}=372 nm (I_1) and λ_{em}=392 nm (I_3) at different LG18 concentrations.

LG18 Concentration (μM)	I_1/I_3	LG18 Concentration (μM)	I_1/I_3	LG18 Concentration (μM)	I_1/I_3
0	1.124	8.04	1.087	12.9	1.051
0.24	1.124	8.67	1.082	16.7	1.036
0.47	1.123	9.28	1.073	17.6	1.034
2.25	1.116	10.1	1.071	18.3	1.031
4.19	1.108	10.7	1.066	19.0	1.026
5.88	1.099	11.2	1.064	20.4	1.014
7.36	1.092	11.7	1.060	21.0	1.009
